# Integrating Brain Imaging Volumetrics and Quantitative Pupillometry for Predicting Neurologic Deterioration after Large Hemispheric Stroke

**DOI:** 10.21203/rs.3.rs-7207393/v1

**Published:** 2025-09-17

**Authors:** Yili Du, Leigh Ann Mallinger, Allyson L. Reinert, Stefanos Chatzidakis, Nawal J. Ibrahim, Gabriella Wirth, Atul Kumar, Amrit Avula, Huimin Cheng, David M. Greer, Rajat Dhar, Charlene Ong

**Affiliations:** Boston University School of Public Health; BMC: Boston Medical Center; BMC: Boston Medical Center; Brigham and Women's Hospital; Boston University School of Public Health; BMC: Boston Medical Center; Washington University School of Medicine in Saint Louis: Washington University in St Louis School of Medicine; Washington University School of Medicine in Saint Louis: Washington University in St Louis School of Medicine; Boston University School of Public Health; Boston University School of Medicine: Boston University Chobanian & Avedisian School of Medicine; Washington University School of Medicine in Saint Louis: Washington University in St Louis School of Medicine; Boston University Medical Campus

**Keywords:** cerebrospinal fluid, pupil, stroke, cerebral edema, prognosis, biomarkers

## Abstract

**Background:**

Cerebral edema is a life-threatening complication of large ischemic stroke. Imaging assessment of global and hemispheric cerebrospinal fluid (CSF) volumetrics quantifies edema progression, while quantitative pupillometry provides real-time bedside assessment of neurologic decline. However, the relationship between the two and their combined value for predicting neurologic deterioration remains unclear.

**Methods:**

We conducted a retrospective study of patients with large middle cerebral artery strokes admitted to Boston Medical Center between 2019 and 2024. Eligible patients had ≥1 head CT and ≥3 pupillometry measurements. Total and hemispheric CSF volumes were extracted using an automated image analysis pipeline. Average pupillometry variables, including the Neurological Pupil index (NPi) and dilation velocity, were aligned to imaging within ±1 hour and within the subsequent 24-hours of each image. Associations between pupillometry and CSF volumetrics were evaluated using Spearman’s correlations and linear mixed-effects models adjusted for age, sex, and standardized baseline brain volume. Cox proportional hazards models with time-dependent covariates were used to assess the predictive value of CSF and pupillometry markers for time-to-neurologic deterioration. We compared model performance using likelihood ratio tests and time-dependent area under the curve (AUC) metrics.

**Results:**

Seventy-one patients (mean age 66 ±16 years; 59% women) with 249 CT images were included. Pupillometry and CSF measures were significantly correlated in the first 48-hours post-stroke. In adjusted models, lower hemispheric CSF volume ratio was associated with lower NPi (β=1.55, p=0.02) and greater NPi difference (β=−1.53, p<0.01). Thirty-two (46%) of 69 eligible patients experienced neurologic deterioration. Models including CSF volume and pupillometry outperformed those with pupillometry only (AUC 83.5% v. 81.0%; χ^2^=4.63, *p*=0.03).

**Conclusions:**

Pupillometry and imaging-derived CSF volumetrics are temporally aligned biomarkers that improve prediction of neurologic deterioration, supporting their complementary roles in monitoring cerebral edema.

## Introduction

Life threatening cerebral edema can affect up to 30% of patients with large middle cerebral artery (MCA) stroke.^1^ Despite management advances,^2^ critical gaps remain in monitoring and therapeutic strategies^3^ due to the lack of objective, accurate, non-invasive bedside methods to predict and monitor swelling and mass effect. Quantitative pupillometry has been shown to improve prediction of neurologic deterioration, an important and potentially preventable complication linked to overall outcomes, compared to standard clinical tools such as the EDEMA score.^1^ Similarly, reductions in total and hemispheric CSF volume on follow-up CT imaging have been associated with progressive radiographic edema after stroke. These imaging-based volumetric biomarkers have also outperformed the EDEMA score in predicting malignant cerebral edema.^4^ What remains uncharacterized is the relation between quantitative pupillometry and worsening radiographic edema, as measured by imaging-based volumetrics, and whether combining these distinct non-invasive biomarkers improves prediction of neurologic deterioration and outcomes following large ischemic stroke. To address this gap, our aims were the following: 1) Evaluate the association between CSF-based imaging volumetric measures of edema and pupillometry features; and 2) Assess the predictive value of integrating pupillometry and CSF volumetric data to predict neurologic deterioration.

## Methods

### Study Design and Participants

**Figure 1:**
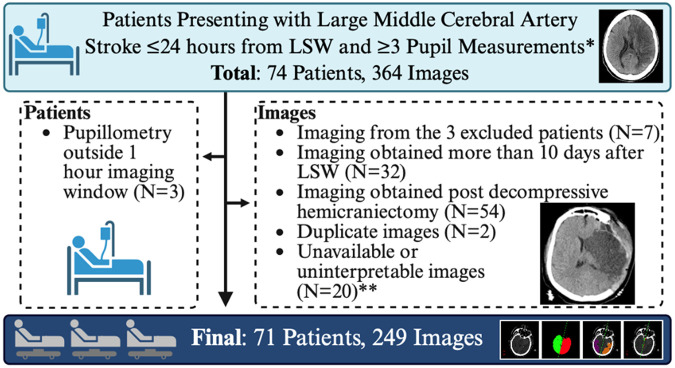
Patient and Image Eligibility Flow Chart *Boston Medical Center ICU patients (2019-2024).**Imaging exclusions: uninterpretable image (N=13), segmentation failure (N=4), no axial brain (N=2), no PDF (N=1). Neurological deterioration analysis: 69 patients; 2 excluded for absent pupillometry prior to deterioration. Abb.: LSW=Last Seen Well.

We performed a retrospective single-center observational study of adult patients with large ischemic stroke admitted to the neuroscience intensive care unit at Boston Medical Center between 2019 and 2024, all of whom were enrolled in an ongoing prospectively collected cohort.^1^ All participants had ≥1/2 MCA territory infarct evident on head CT or MRI within 72 hours of admission, as adjudicated by previously published methods,^1^ had at least three quantitative pupil observations, and presented within 24 hours of stroke onset. All participants had one or more CT images during their admission. We excluded images acquired more than 10 days after last seen well, post-hemicraniectomy, duplicative images, or had quality issues that interfered with CSF segmentation. Detailed eligibility criteria are listed in FIGURE 1.

This study was approved by the Boston Medical Center and Boston University Medical Campus Institutional Review Board (H-37699) with a waiver of patient consent, as it was retrospective and did not deviate from standard care. All methods were performed in accordance with the relevant guidelines and regulations set forth by the IRB. We adhered to the Strengthening the Reporting of Observational Studies in Epidemiology (STROBE) guidelines for reporting the study.

### Clinical Data Collection

We collected demographic and clinical information from the electronic medical record, including time last seen well, past medical history, stroke assessment, procedures, and outcomes as previously described.^1^ Additional radiographic features including midline shift and basal cistern effacement were collected by trained team members following the same published protocol.^1^

### Pupillometry Data and Processing

Quantitative pupillometry measurements were obtained by trained nurses using the NeurOptics NPi-300 pupillometer (NeurOptics Inc., Irvine, CA) at intervals of one, two, or four hours, depending on the frequency of neurologic checks ordered per standard care in the neuroscience intensive care unit. Collected pupillometric variables included the Neurological Pupil index (NPi), resting pupil size, and dilation velocity. The NPi scale ranges between 0 and 5. NPi <3 in either eye or pupils size asymmetry (resting pupil size difference >1mm) are considered abnormal according to the manufacturer^5^ and prior studies.^6^ Complete data for NPi and pupil size were available for all patients through the electronic medical record. Dilation velocity data were extracted from individualized SmartGuards, which store detailed pupillometry measurements. We consolidated pupil characteristics between left and right eye including minimum NPi, NPi difference, pupil size difference, and the minimum dilation velocity. Further details are provided in the **Supplementary Methods**.

### Image Analysis

Head CT images were analyzed using a previously validated deep learning-based image analysis pipeline that automatically extracts brain tissue, segments CSF and infarct regions, and divides the brain into two hemispheres using the anatomical mid-sagittal plane.^7^ At each time point, we derived the following imaging metrics: 1) total and hemispheric (ipsilateral and contralateral) CSF volumes, with the hemispheric CSF ratio calculated as the CSF volume in the affected hemisphere divided by that in the contralateral hemisphere; 2) infarct volume, calculated from the segmented infarct region when a hypodense region was visible; 3) total and hemispheric brain parenchymal volumes, calculated as intracranial volume excluding CSF volume. The hemispheric brain ratio was calculated as the brain volume in the affected hemisphere divided by the contralateral hemisphere; 4) net water uptake, calculated as the percentage reduction in CT attenuation (Hounsfield Units) of the lesion compared to the corresponding normal brain tissue;^7^ 5) change in CSF, calculated as the percent change in total CSF volume between baseline and follow-up CT scans; and 6) total intracranial volume. From each patient’s first available CT, we also calculated absolute brain volume, standardized brain volume, and standardized CSF volume by dividing total brain and CSF volumes by total intracranial volume.

### Statistical Analysis

#### Descriptive and Exploratory Correlations

We performed descriptive analyses to summarize patient-level characteristics using counts and proportions for categorical variables and means with standard deviations or medians with interquartile ranges (IQR) for continuous variables, as appropriate. Distributions of baseline CSF volumetric data and their differences across sexes were visualized using histograms. We reported their means and standard deviations.

Pupillometric measures were aligned to volumetric imaging by identifying pupil observations within ±1 hour of the imaging time. From this subset, we derived summary values including: the measurement closest to imaging, average value, minimum or maximum value (to capture “most abnormal” status), as well as binary indicators for clinically significant thresholds (NPi <3 or size difference >1mm). Because imaging is obtained at clinical discretion and not necessarily at the time of worsening edema development, we additionally computed average values of the above variables within 24 hours following each image.

We visualized longitudinal trajectories of CSF volumetric variables using spaghetti plots and reported their distribution stratified by time from last seen well using boxplots. Correlations between pupillometry variables and CSF volumetric measures were assessed using Spearman’s Rank correlation coefficients. We further evaluated the correlations between pupillometry and CSF volumetric data within 0-24, 24-48, 48-72 hours after last seen well.

#### Adjusted Mixed-Effects Models between Pupillometry and CSF Variables

Building on the results of the exploratory correlations, we used mixed-effects linear models with random intercept to investigate the association of pupil characteristics and CSF volume variables. Our primary outcome included the minimum observed NPi value ±1 hour of imaging. To address the fact that clinical imaging is not necessarily obtained at the time of observed pupillary change, we also examined the average NPi within 24 hours post-imaging (NPi_24post_). Additional outcomes included NPi measured in time closest to the imaging study, maximum NPi difference between eyes, and minimum dilation velocity within ±1 hour of imaging. Our primary exposure was CSF volume ratio, a validated marker of increasing mass effect.^8^ We also explored the association with total CSF volume.

To satisfy the normality assumption of residuals for linear regression, we transformed skewed CSF ratio data using the cube root of the original ratio measurement subtracted from 1.0 (the maximal value), with higher values representing more edema when appropriate.^9^ Models were adjusted for age, sex, and baseline standardized brain volume (total parenchymal volume/total intracranial volume) as fixed effects. To account for within-subject correlation arising from repeated measurements, we included random intercepts for each subject in the mixed-effects models. We reported beta estimates and p-values. To account for multiple hypothesis testing across various exposure-outcome pairs, we used the Benjamini-Hochberg procedure to determine the significance.^10^

#### Neurologic Deterioration Prediction Models

To assess the added value of CSF metrics in predicting time-to-neurologic deterioration, we developed Cox proportional hazard models, treating longitudinal pupillometry including NPi and dilation velocity (the optimal predictors identified in prior work),^1^ and the time-aligned CSF volumetric data as time-dependent covariates, as additions to the EDEMA score.^11^ The analysis was performed in a subgroup of patients who did not have neurologic deterioration prior to the time of pupillometry.

Baseline models included established predictors such as the EDEMA score alone (Model 1), and EDEMA plus pupillometry features (Model 2). We then added CSF volume ratio (Model 3) and total CSF volume (Model 4) to assess incremental predictive performance. We used all available pupillometric measurements within 10 days of last seen well and the closest imaging associate with each pupillometric measurement in these models. To quantify the predictive value introduced by the CSF metrics, we compared nested prediction models using likelihood ratio (χ^2^) tests and compared the model discrimination among all models using time-dependent area under the receiver operating characteristic curves (AUCs) averaged over the observation window.

To account for censoring and repeated measurements in time-to-event analyses, we used inverse probability of censoring weighting (IPCW) ^12^ when calculating time-dependent AUCs. To assess model generalizability and reduce the risk of overfitting, we implemented a leave-one-out cross-validation framework, ^13^ in which each patient’s outcome was predicted using a model trained on all other patients.

Finally, we evaluated extended alternative models that included fixed variables including baseline glucose and standardized brain volume, and time-dependent variables including midline shift, pupillometry, and either CSF volume ratio (Model 5) or total CSF volume (Model 6). These models aimed to improve the EDEMA model using more quantified metrics that correlate with its original constructs. We compared performances of Model 5 and Model 6 with the others using both averaged time-dependent AUCs and Akaike Information Criterion (AIC) and Bayesian Information Criterion (BIC).^14^

All analyses were conducted in R (version 4.5.0). Statistical significance was defined as a two-sided p-value < 0.05 unless otherwise indicated.

## Results

### Study Cohort Characteristics

A total of 364 head CTs from 71 patients were evaluated. After exclusions (N=115), due to missing paired pupillometry data within1 hour of imaging (N=7), delayed imaging (N=32), post-decompressive hemicraniectomy (N=54), image duplication (N=2) or unavailability/poor quality imaging (N=20), 249 images were included in the final analysis (FIGURE 1). Patient population characteristics are included in TABLE 1. Average age was 66 ±16 years, with 59% (N=42) female. Stroke occurred on the right side in 46.5% (N=33) of patients. Patients underwent a median of 3 [IQR 2–5] head CTs and 170 [IQR 92–234] total pupillometry measurements over 10 days (TABLE 1).

**Figure 2:**
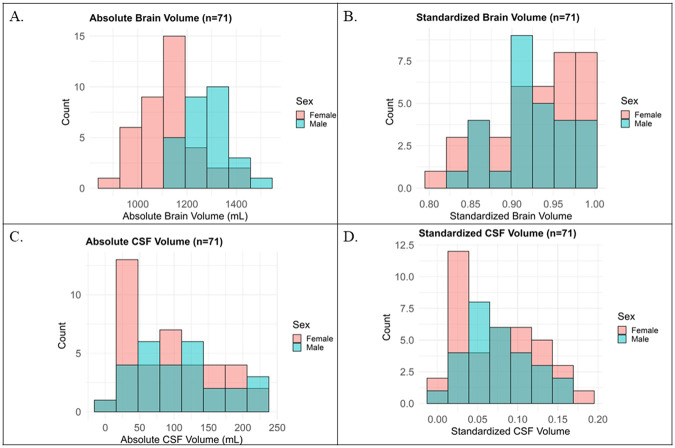
Baseline Volumetric Data by Sex Absolute brain volume (A) was lower in females compared to males (1112±115 mL v. 1283±86 mL). standardized brain volume (total brain volume divided by total cranial volume, B) was similar across females (0.92±0.05) and males (0.92±0.04). CSF volume (C) also did not significantly differ between females (92±63 mL) and males (106±63mL). D) Standardized CSF volume (total CSF volume divided by total cranial volume) also showed no meaningful sex-based difference: females (0.08±0.05) and males (0.08±0.04). Abb.: CSF=Cerebrospinal Fluid.

Our consolidated pupillometric dataset consisted of 189 and 249 observations, respectively, for NPi and NPi_24post_. The average NPi around the time of imaging was 3.82±1.45 at 24 hours, 4.13±1.13 at 48 hours, and 4.16±0.95 at 72 hours after last seen well. The average NPi over 10 days after last seen well was 4.00±1.20 (TABLE 1). The average baseline infarct volume on the first available image was 90±86 mL (N=69), and the maximum infarct volume was 172±90 mL. Average standardized brain volume at baseline was 0.93±0.05 (N=71), and average standardized CSF volume at baseline was 0.07±0.05 (N=71) (TABLE 1), with similar values observed across sexes (FIGURE 2). The average CSF volume ratio was 0.61±0.23 at 24 hours, 0.41±0.22 at 48 hours, and 0.41±0.18 at 72 hours after last seen well. The 10-day average CSF volume ratio was 0.49±0.25.

**Figure 3:**
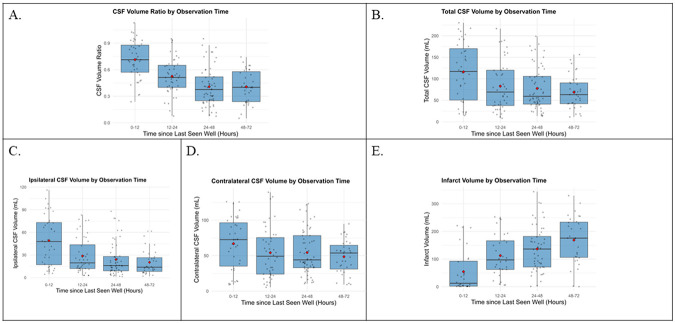
Temporal Trends in CSF and Infarct Volume CSF volume ratio (A) and total CSF volume (B) declined over the 48 hours after Last Seen Well, with ipsilateral CSF volume (C) declining as expected, and contralateral CSF volume (D) remainign relatively stableafter 12-24 hours. Inversly, infarct volume (E) increased over the same time period. Abb.: CSF=Cerebrospinal Fluid.

Over the course of hospitalization, ipsilateral CSF volume and CSF volume ratio declined, particularly in the 72 hours after stroke, while infarct volume increased (FIGURE 3**, Supplementary FIGURE 1)**. Contralateral CSF, total CSF, brain volume ratio, and net water uptake showed no consistent trend (FIGURE 3**, Supplementary FIGURES 1-2)**. In 86% of patients with at least two CT scans (N=61), CSF volume ratio and ipsilateral CSF decreased, and infarct volume increased compared to the baseline scan (**Supplementary FIGURE 3**). Spaghetti plots from 67 patients with imaging within 72 hours demonstrate these trends (**Supplementary FIGURE 4**).

### Quantitative Pupillometry and CSF Volume Correlations

Using all available pupillometric and CSF variable pairs, we did not observe significant spearman correlations between the NPi and CSF volume ratio (**Supplementary TABLE 1**). The spearman correlation between standardized brain volume in patients with reactive pupils at the first available image and NPi or dilation velocity was not statistically significant (**Supplementary FIGURE 5)**. However, when stratified by time, we found that CSF volume ratio and NPi were more correlated in 24-36 hours after last seen well (r_s_=0.43, p=0.01). We also observed significant correlations between NPi_24post,_ NPi difference, and dilation velocity with total CSF volume in the first 48 hours (TABLE 2).

### Adjusted Models of CSF Metrics with Pupillometric Characteristics

Adjusting for multiple hypotheses, we observed significant associations in our adjusted models of CSF Volume Ratio and NPi (b=1.55, p=0.02), NPi_24post_ (b=−0.76, p=0.01) and NPi Difference (b=−1.53, p<0.01). Total CSF was significantly associated with NPi_24post_ (b=0.004, p<0.01) (TABLE 3).

### Added Predictive Value of CSF Metrics for Neurologic Deterioration

**Figure 4:**
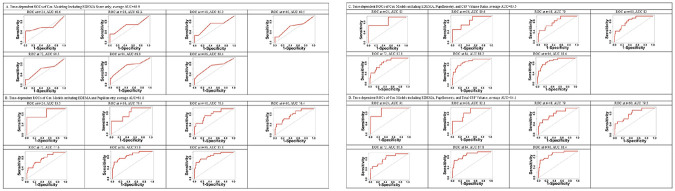
Time-dependent Receiver Operating Characteristic Curve for Cox models of Neurological Deterioration in Each 12-hour Interval Time-dependent ROC curves for Cox models predicting time-to-neurologic deterioration, adjusted by EDEMA score (fixed variable), pupillometry (NPi and dilation velocity) and CSF volumetric data (time-dependent covariates) in each 12-hour interval. Panel A: EDEMA; Panel B: EDEMA and pupillometry; Panel C: EDEMA, pupillometry, and CSF volume ratio; Panel D: EDEMA, pupillometry, and total CSF volume. Overall average AUCs of models displayed in panels C and D (including CSF volumetric data) were higher compared to models in panels A and B (not including CSF volumetric data). Abb.: AUC=Area Under Curve; CSF=Cerebrospinal Fluid; EDEMA=Enhanced Detection of Edema in Malignant Anterior Circulation Stroke; NPi=Neurological Pupil Index; ROC=Receiver Operating Characteristic.

For our analysis of neurologic deterioration, 69 patients were eligible, as two patients experienced neurologic deterioration prior to first available pupil measurement. Of these 69 patients, 32 (46%) experienced neurologic deterioration. Inclusion of pupillometry and CSF volumetric metrics improved discrimination (average time-dependent AUC 83.5%) over both Model 1 (the baseline EDEMA score) and Model 2 (baseline EDEMA + pupillometry) (average time-dependent AUC 83.5 v. 63.9% v. 81.0%) (FIGURE 4). Using Leave-One-Out Cross-Validation to further reduce overfitting and assess generalizability, the CSF-inclusive model maintained superior discriminative performance compared to the pupillometry-only model (AUC 81.2% v. 79.2%) **(Supplementary FIGURE 6)**.

There was a significant improvement of model fit between the model that included total CSF volume over EDEMA + pupillometry (Model 4 v. Model 2; χ^2^=4.63, degree of freedom=1, p=0.03) and a trend to improve model fit when adding CSF volume ratio (p=0.10) (TABLE 4) Models 5 and 6 (including quantitative markers of the EDEMA score) discriminated similarly with AUCs of 81.0% and 82.1%, respectively, but with higher AIC and BIC values compared to Models 2–4 (TABLE 4**, Supplementary FIGURES 7-8**).

## Discussion

In this study, we found that bedside quantitative pupillometry and CT-based volumetric markers of worsening mass effect are correlated during the period of highest risk for edema progression. Notably, this correlation was absent early in admission and when values were averaged over the first several days, suggesting that both pupillometry and CSF volume are context-dependent markers of secondary injury. We also observed that combining pupillometry with CSF volumetrics improved the discrimination of neurologic deterioration, particularly when compared to using baseline measures alone. These findings support their use as complementary biomarkers that, when integrated, may improve prediction of a patient’s cumulative risk for imminent edema-related neurologic decline after stroke.

Prior work has examined the association between quantitative pupillometry and midline shift.^15,16^ However, the full neuroanatomic mechanisms underlying decreased pupil reactivity, which is not solely related to midline shift, remains poorly understood.^17,18^ The development of automated CSF segmentation algorithm now enables more detailed investigation of anatomic correlates of pupillary dysfunction. CSF dynamics are a critical but underexplored component of intracranial compliance and pressure regulation in large ischemic stroke. In the early stages of cerebral edema, CSF redistribution, particularly as it relates to sulcal effacement and reduced ventricular and cisternal volumes, has been proposed as a compensatory mechanism to buffer rising intracranial volume and delay clinical deterioration.^19^ Dynamic reductions in total and hemispheric CSF volume have been associated with the development of malignant edema in several studies.^8,20^ Building on this evidence, our aim was to characterize radiographic markers of malignant edema that precede more overt signs such as midline shift.

In this study, our observed interaction between time, pupillometry, and CSF volume highlights the importance of context and timing when interpreting pupil reactivity. Given that pupil dynamics are influenced by numerous factors, including ambient light,^21^ cognitive activity, and medications,^22,23^ minor quantitative fluctuations in reactivity do not necessarily indicate worsening mass effect. However, when assessed within the appropriate clinical window, a meaningful association emerges between anatomical evidence of edema and pupillometric changes. Subtle pupillary changes may precede overt clinical deterioration, providing an opportunity for more timely intervention, as we demonstrated that reductions in CSF volume, which can even precede midline shift^15^ are associated with alterations in pupillometry during the expected time window of worsening cerebral edema. We suspect that sulcal effacement and subsequent injury producing mass effect precedes changes in pupillometry, as suggested by the stronger association with average pupillometry over the subsequent 24 hours. We posit that the strongest associations between lower CSF volume and pupillometric variables arise from increasing sulcal and ventricular effacement as mass effect rises. Although the CSF ratio has been associated with malignant edema,^19^ its reliability in cohorts with midline shift may be limited by ipsilateral versus contralateral miscalculations due to anatomic distortions in patients with severe edema.

Our time-dependent models for neurologic deterioration suggest that combining radiographic and pupillometric features may augment dynamic risk assessment beyond standard risk scores or even using either new biomarker alone. The highest discrimination for imminent neurologic deterioration was achieved by a model that included the standard EDEMA score along with both pupillometry and CSF volume data, demonstrating significantly better fit compared to a model that included only pupillometry and the EDEMA score. This combined model also outperformed one incorporating the CSF volume ratio alone, particularly in the 12–24-hour subgroup (AUC 91.0 v. 83.0%) (FIGURE 4).

We observed CSF volume declines in a cohort of large strokes over the peak swelling window (24-72 hours), consistent with other studies.^8^ We also found that excluding NPi values of 0 (signifying patients with early extensive mass effect) eliminated the correlation between pupillometry and standardized brain volume at baseline, suggesting that pupillary changes signify increasing mass effect independently of cerebral atrophy and inversely total CSF volume **(Supplemental FIGURE 5)**.

Importantly, these results build on previous studies demonstrating that serial quantitative biomarkers can predict imminent neurologic events better than existing risk scores.^1,4,24^ Our findings support integrating simple bedside and radiographic features into clinical assessments to evaluate whether they improve early recognition of deterioration and, ultimately, patient outcomes compared to standard care alone. While CSF segmentation is not yet standard in clinical practice, advancing technologies are rapidly paving the way for routine use of quantified radiographic metrics. Our results are preliminary; however, they lay the groundwork for rigorous, evidence-based variable selection to optimize the sensitivity and specificity of dynamic predictive models. Once optimized models are validated, they can serve multiple purposes in 1) facilitating more timely treatment, minimizing secondary brain injury and 2) improving patient selection for future therapeutic trials.

We recognize several limitations. Our study was retrospective and single center, limiting its generalizability. Since follow-up brain imaging was performed at the discretion of the clinical team, directly correlating radiographic signs of increasing mass effect with changes in pupil reactivity was challenging and subject to some bias. Missing values in 14 patients for dilation velocity, particularly if not missing at random, may introduce bias into the Cox regression model, potentially distorting hazard ratio estimates and reducing the overall statistical power. While statistical adjustments were made for known confounders, the possibility of residual confounding from unmeasured variables remains. Additionally, the relatively small sample size constrained our ability to adjust for multiple covariates without risking model overfitting and limited our statistical power for subgroup analyses. Automated CSF segmentation can fail in the presence of motion artifacts, variable slice thickness, or post-craniectomy anatomy, leading to exclusion of the most severe or technically challenging cases and potential selection bias. Because the pipeline was developed and validated at a single center using specific scanners and reconstruction kernels, its accuracy and reproducibility may diminish elsewhere without recalibration.

Despite these limitations, our study has several strengths. It is, to our knowledge, the first study to systematically examine the relationship between quantitative pupillometry and volumetric brain imaging longitudinally after stroke. The use of standardized protocols and equipment for data collection enhances the objectivity and reproducibility of pupillometry measurements. By incorporating predefined confounders, we sought to minimize bias and strengthen the validity of our results. Moreover, the application of advanced statistical approaches, including linear mixed-effects models and Cox proportional hazards models with time-varying covariates, enabled a detailed analysis of how pupillary dynamics relate to CSF volumetrics over time.

Our automated analysis of CSF displacement offers several advantages, including reproducible and scalable measures of global and hemisphere-specific edema progression that can be automatically extracted from routinely acquired head CTs. This leads to reduced inter-rater variability and requires no additional imaging, allowing seamless integration into clinical workflows. It complements the bedside assessments that pupillometry affords, by providing an imaging-based assessment of intracranial compliance, capturing compensatory shifts in intracranial compliance that precede midline shift and may occur before pupillary changes.

## Conclusions

Pupillometry and CSF volumetric analysis are complementary biomarkers for tracking evolving cerebral edema from mass effect after ischemic stroke. Future prospective studies are needed to validate these models of neurologic deterioration that integrate these promising quantitative biomarkers within clinical workflows and test their ability to assist with clinical decision-making.

## Supplementary Material

This is a list of supplementary files associated with this preprint. Click to download.

NCCSTROBEChecklist7.23.25.docx

NCCSupplementaryMaterial07.24.25.docx

## Figures and Tables

**Table 1.** Demographic and Clinical Characteristics

Variable	All N=71
**Demographics**	
Age, years	66.4 ± 16.2
Female	42 (59.2%)
Race	-
White	21 (29.6%)
Black	18 (25.4%)
Asian	4 (5.6%)
Other^a^	28 (39.4%)
Ethnicity	-
Hispanic or Latino	12 (16.9%)
Not Hispanic or Latino	46 (64.8%)
Not reported and unknown	13 (18.3%)
**Past Medical History**	-
Atrial Fibrillation	19 (26.8%)
Hypertension	43 (60.6%)
Prior Stroke	9 (12.7%)
**Stroke Assessment**	-
Right Sided Stroke	33 (46.5%)
NIHSS scores at presentation	19.8 ± 6.5
Admission ASPECTS	5.7 ± 2.9
Hemorrhagic Transformation	42 (59.2%)
Petechial only	26 (36.6%)
Parenchymal	19 (26.2%)
Min Glasgow Coma Scale	8.5 ± 3.1
Max Midline Shift, mm	6.4 ± 4.8
**Measurement Count**	
Image Count	3 [2-5]
Pupil Count	170 [92-234]
**Volumetric Data**	-
Baseline CSF Volume, mL	98 ± 63
Baseline Standardized Brain Volume	0.93 ± 0.05
Max Infarct Over All Scans for Each Patients, mL	171.57 ± 89.9
Baseline Infarct Volume, mL (n=69)	90 ± 86
Baseline Standardized CSF Volume	0.07 ± 0.05
CSF Volume Ratio Post Last Seen Well	
10 days	0.49 ± 0.25
24 hours	0.61 ± 0.23
48 hours	0.41 ± 0.22
72 hours	0.41 ± 0.18
**Neurological Pupil Index (NPi)**	-
10 Days Post Infarct	4.00 ± 1.20
NPi Peri-Image Post Last Seen Well	-
24 hours	3.82 ± 1.45
48 hours	4.13 ± 1.13
72 hours	4.16 ± 0.95
**Median Timing Data**	-
Time from LSW to First Image (hours)	11 [9-17]
Time from LSW to First Pupil (hours)	10 [7-14]
Length of Stay (days)	7.5 [5-14]
**Procedure/Treatment**	-
Intravenous Thrombolysis^b^	14 (19.7%)
Mechanical Thrombectomy	56 (78.9%)
TICI scale ≥ 2b^c^	43 (60.6%)
Osmotic Therapy	30 (42.3%)
Decompressive Hemicraniectomy	19 (26.8%)
Withdrawal of Life Sustaining Therapy	28 (39.4%)
**Outcomes**	-
Neurologic Deterioration due to Cerebral Edema	26 (36.6%)
Death at Discharge	22 (31%)

a Other Races, includes American Indian, Alaska Native, Native Hawaiian, Pacific Islander, Not Recorded, Not Given or Unknown.

b Tissue Plasminogen Activator (tPA) before 2023 and Tenecteplase (TNK) since 2023.

c TICI ^3^ 2b includes TICI 2b, 2c and 3, which corresponds to either slow or normal complete visualization of the vasculature. Abb.: ASPECTS=Alberta Stroke Program Early CT Score; CSF=Cerebral Spinal Fluid; LSW=Last Seen Well; NIHSS=National Institutes of Health Stroke Scale; NPi=Neurological Pupillary index; TICI=Thrombolysis in Cerebral Infarction.

**Table 2.** Correlation Between Total CSF Volume and Quantitative Pupillometry Around Imaging by Time of Last Seen Well

	Quantitative Pupillometry									
Time Window of Last Seen Well (hours)	0-24h		24-48h		24-36h		36-48h		48-72h	
Spearman Correlation and P value	*r_s_*	*p*	*r_s_*	*p*	*r_s_*	*p*	*r_s_*	*p*	*r_s_*	*p*
	NPi_24post_									
CSF Volume Ratio	0.04	0.72	0.22	0.10	0.34	0.04*	0.00	0.99	0.09	0.61
Total CSF Volume	Minimum NPi									
	0.33	0.01*	0.33	0.02*	0.43	0.01*	0.14	0.58	0.15	0.45
	Average NPi									
	0.31	0.02*	0.32	0.03*	0.41	0.02*	0.12	0.64	0.02	0.92
	Minimum Ipsilateral NPi									
	0.37	0.00*	0.33	0.02*	0.43	0.01*	0.07	0.79	0.07	0.72
	Minimum Dilation Velocity									
	0.33	0.02*	0.32	0.04*	0.38	0.05	0.09	0.72	0.05	0.83
	Maximum NPi Difference									
	−0.28	0.03*	−0.18	0.21	−0.10	0.58	−0.20	0.42	−0.32	0.10
	Maximum Pupil Size Difference									
	−0.12	0.38	0.09	0.56	0.15	0.41	−0.02	0.94	−0.26	0.18
	NPi_24post_									
	0.29	0.01*	0.32	0.01*	0.43	0.01*	0.17	0.44	0.20	0.26
	NPi <3 **									
	−0.47	0.00*	−0.18	0.17	−0.23	0.17	−0.06	0.78	−0.25	0.16
	Size Difference >1mm **									
	−0.27	0.04*	0.07	0.64	0.07	0.70	0.05	0.86	−0.12	0.57

All summary variables are evaluated in ±1 hour of imaging, except NPi_24post_ (average NPi 24 hours post imaging).

Data are presented as Spearman's rank correlation coefficient [r_s_] and p value. Asterisk indicates significance of p value(p<0.05). ** Point-biserial method was used for the binary variable. Abb.: NPi=Neurological Pupillary index.

**Table 3.** Adjusted Models of Pupillometry and CSF Variables

Pupillometry Variables	Total CSF		CSF Volume Ratio**	
	*β*	*p*	*β*	*p*
Closest NPi	0.01	0.07	1.37	0.05
Minimum NPi	0.005	0.09	1.55	0.02*
Maximum NPi Difference	−0.001	0.57	−1.53	0.003*
NPi_24post_	0.003	0.002*	−0.76	0.01*

All summary variables are evaluated in ±1 hour of imaging, except NPi_24post_ (average NPi 24 hours post imaging). All models were adjusted for age, sex, and baseline standardized brain volume. *P value significance determined by Benjamini-Hochberg procedure. Asterisk indicates an adjusted p value of < 0.05. **To satisfy the residual assumption for linear regression, we transformed skewed CSF ratio data using the cube root of the original ratio measurement subtracted from 1.0 (the maximal value), with higher values representing more edema when appropriate. Abb.: CSF=Cerebrospinal Fluid; NPi=Neurological Pupil Index.

**Table 4.** Comparison of Cox Models for Neurologic Deterioration

Cox Models^a^	Likelihood Ratio Test between Nested Models		Average AUC	AIC	BIC
Covariates	χ^2^	p			
Static Model					
Model 1: EDEMA score only			63.90	222.50	223.96
Baseline Model					
Model 2: EDEMA score + NPi_t_ + DV_t_			81.00	128.09	131.50
Primary Models					
Model 3: EDEMA score + NPi_t_ + DV_t_+ CSF Volume Ratio_t_	2.67	0.10	83.50	127.42	131.96
Model 4: EDEMA score + NPi_t_ + DV_t_ + Total CSF Volume_t_	4.63	*0.03	84.10	125.46	130.00
Exploratory Alternative Models					
Model 5: Baseline Glucose+ Baseline Standardized Brain Volume + NPi_t_ + DV_t_ + CSF Volume Ratio_t_			81.00	130.85	137.39
Model 6: Baseline Glucose+ Baseline Standardized Brain Volume + NPi_t_ + DV_t_+ Total CSF Volume_t_			82.10	130.31	136.86

a 55 of 69 patients had dilation velocity and CSF data available, all model comparisons were performed in the same reduced dataset (N=55 patients).

b The p-values from Likelihood Ratio test evaluated whether nested Cox model including CSF predictor (Model 3, Model 4) has superior fit than the baseline model (Model 2) at two-sided significance level of 0.05.

c Asterisk indicates a p value of <0.05. Abb.: CSF=Cerebrospinal Fluid; DV=dilation velocity; EDEMA=Enhanced Detection of Edema in Malignant Anterior Circulation Stroke; NPi=Neurological Pupil Index.
